# Supplementary material for: Antibodies to repeat-containing antigens in Plasmodium falciparum are exposure-dependent and short-lived in children in natural malaria infections
Source: eLife. 2023 Feb 15;12:e81401. doi: 10.7554/eLife.81401 (PMC10005774; doi:10.7554/eLife.81401)
Supplement: Supplementary file 2. [file elife-81401-supp2.zip › Supplementary_File2_Top40SeropositiveProteins_literature.docx]

**Supplementary table 2 – Top Proteins with highest seropositivity (>38%) in the cohort**

| **Known targets of protective antibodies in humans or animal models** | | **No evidence as targets of protective antibodies** | |
| --- | --- | --- | --- |
| *Characterized in high-resolution in this study* | *Previously*  *well-characterized* | *Characterized in high-resolution in this study* | *Previously*  *well-characterized* |
| PHISTc(PF3D7_0801000) ^1^  MSP10^2^  Rh2a ^3,4^  Rh2b ^3,4^  EMP3 ^5^  RON2^6^  DBLMSP^7^ | Ag332 ^8–10^  LSA3^11^  STARP^12^  GLURP^13–15^  MSP4^16,17^  MSP2^18–20^  EBA175 ^21,22^  KAHRP^23^  MSP1^24^  PF11-1^25^  RIFIN ^26,27^  STEVOR ^28^  PfEMP1^29,30^ | SURFINs  REX1  SBP1  TREP  PF3D7_0424700(FIKK Ser/Thr Kinase)  PF3D7_0511500 (RNA pseudouridylate synthase)  PTP3  RON4  LISP2  PTP4  CRMP4  PF3D7_0628100  PF3D7_1023100(putative dyenin heavy chain)  PF3D7_0907200(putative HECT domain-containing protein 1)  PFIT_1002300  PFIT_0522600  CRA(EXP1) | FIRA ^31^  MESA ^32,33^  LSA1 (Protective T cell responses - ^34^) |

1. Nagaoka, H. *et al.* Characterization of a Plasmodium falciparum PHISTc protein, PF3D7_0801000, in blood- stage malaria parasites. *Parasitol. Int.* **80**, 102240 (2021).

2. Nagaoka, H. *et al.* The N-Terminal Region of Plasmodium falciparum MSP10 Is a Target of Protective Antibodies in Malaria and Is Important for PfGAMA/PfMSP10 Interaction. *Front. Immunol.* **10**, 2669 (2019).

3. Sahar, T. *et al.* Plasmodium falciparum reticulocyte binding-like homologue protein 2 (PfRH2) is a key adhesive molecule involved in erythrocyte invasion. *PLoS One* **6**, 17102 (2011).

4. Triglia, T. *et al.* Plasmodium falciparum Merozoite Invasion Is Inhibited by Antibodies that Target the PfRh2a and b Binding Domains. *PLOS Pathog.* **7**, e1002075 (2011).

5. Grüner, A. C. *et al.* The Plasmodium falciparum knob-associated PfEMP3 antigen is also expressed at pre-erythrocytic stages and induces antibodies which inhibit sporozoite invasion. *Mol. Biochem. Parasitol.* **112**, 253–261 (2001).

6. Srinivasan, P. *et al.* Immunization with a functional protein complex required for erythrocyte invasion protects against lethal malariia. *Proc. Natl. Acad. Sci. U. S. A.* **111**, 10311–10316 (2014).

7. Chiu, C. *et al.* Antibodies to the Plasmodium falciparum Proteins MSPDBL1 and MSPDBL2 Opsonize Merozoites, Inhibit Parasite Growth, and Predict Protection From Clinical Malaria. *J. Infect. Dis.* **212**, 406–415 (2015).

8. Udomsangpetch, R., Aikawa, M., Berzins, K., Wahlgren, M. & Perlmann, P. *Cytoadherence of knobless Plasmodium falciparum-infected erythrocytes and its inhibition by a human monoclonal antibody LETTERS TO NATURE*. *J. 1 Pharm. Pharmac* vol. 338 (1989).

9. Wåhlin, B. *et al.* Involvement of Pf155/RESA and cross-reactive antigens in Plasmodium falciparum merozoite invasion in vitro. *Infect. Immun.* **60**, 443–449 (1992).

10. Moll, K. *et al.* A Novel DBL-Domain of the P. falciparum 332 Molecule Possibly Involved in Erythrocyte Adhesion. *PLoS One* **2**, e477 (2007).

11. Morita, M. *et al.* Immunoscreening of Plasmodium falciparum proteins expressed in a wheat germ cell-free system reveals a novel malaria vaccine candidate. *Sci. Rep.* **7**, 1–8 (2017).

12. Fidock, D. A. *et al.* Plasmodium falciparum sporozoite invasion is inhibited by naturally acquired or experimentally induced polyclonal antibodies to the STARP antigen. *Eur. J. Immunol.* **27**, 2502–2513 (1997).

13. Theisen, M. *et al.* The Glutamate-Rich Protein (GLURP) of Plasmodium falciparum Is a Target for Antibody-Dependent Monocyte-Mediated Inhibition of Parasite Growth In Vitro. *Infect. Immun.* **66**, 11 (1998).

14. Kana, I. H. *et al.* Naturally acquired antibodies target the glutamate-rich protein on intact merozoites and predict protection against febrile malaria. *J. Infect. Dis.* **215**, 623–630 (2017).

15. Jepsen, M. P. G. *et al.* The malaria vaccine candidate GMZ2 elicits functional antibodies in individuals from malaria endemic and non-endemic areas. *J. Infect. Dis.* **208**, 479–488 (2013).

16. Goschnick, M. W., Black, C. G., Kedzierski, L., Holder, A. A. & Coppel, R. L. Merozoite surface protein 4/5 provides protection against lethal challenge with a heterologous malaria parasite strain. *Infect. Immun.* **72**, 5840–5849 (2004).

17. Reiling, L. *et al.* Targets of complement-fixing antibodies in protective immunity against malaria in children. *Nat. Commun.* **10**, 1–13 (2019).

18. Stubbs, J. *et al.* Strain-transcending Fc-dependent killing of Plasmodium falciparum by merozoite surface protein 2 allele-specific human antibodies. *Infect. Immun.* **79**, 1143–1152 (2011).

19. Osier, F. H. A. *et al.* Opsonic phagocytosis of Plasmodium falciparum merozoites: Mechanism in human immunity and a correlate of protection against malaria. *BMC Med.* **12**, 108 (2014).

20. McCarthy, J. S. *et al.* A Phase 1 Trial of MSP2-C1, a Blood-Stage Malaria Vaccine Containing 2 Isoforms of MSP2 Formulated with Montanide® ISA 720. *PLoS One* **6**, e24413 (2011).

21. Jiang, L. *et al.* Evidence for erythrocyte-binding antigen 175 as a component of a ligand-blocking blood-stage malaria vaccine. *Proc. Natl. Acad. Sci. U. S. A.* **108**, 7553–7558 (2011).

22. Chen, E., Paing, M. M., Salinas, N., Sim, B. K. L. & Tolia, N. H. Structural and Functional Basis for Inhibition of Erythrocyte Invasion by Antibodies that Target Plasmodium falciparum EBA-175. *PLoS Pathog.* **9**, e1003390 (2013).

23. Carlson, J., Holmquist, G., TAYLORf, D. W., Perlmann, P. & Wahlgren, M. *Antibodies to a histidine-rich protein (PfiRPi) disrupt spontaneously formed Plasmodium falciparum erythrocyte rosettes*. *Proc. Nati. Acad. Sci. USA* vol. 87 (1990).

24. Blank, A. *et al.* Immunization with full-length Plasmodium falciparum merozoite surface protein 1 is safe and elicits functional cytophilic antibodies in a randomized first-in-human trial. *npj Vaccines* **5**, 1–15 (2020).

25. Feng, Z. *et al.* Pfs2400 can mediate antibody-dependent malaria transmission inhibition and may be the Plasmodium faliciparum 11.1 gene product. *J. Exp. Med.* **177**, 273–281 (1993).

26. Xie, Y. *et al.* Structural basis of malarial parasite RIFIN-mediated immune escape against LAIR1. *Cell Rep.* **36**, 109600 (2021).

27. Tan, J. *et al.* A LAIR1 insertion generates broadly reactive antibodies against malaria variant antigens. *Nat. 2015 5297584* **529**, 105–109 (2015).

28. Niang, M. *et al.* STEVOR is a plasmodium falciparum erythrocyte binding protein that mediates merozoite invasion and rosetting. *Cell Host Microbe* **16**, 81–93 (2014).

29. Bull, P. C. & Abdi, A. I. The role of PfEMP1 as targets of naturally acquired immunity to childhood malaria: prospects for a vaccine. *Parasitology* **143**, 171 (2016).

30. Tessema, S. K. *et al.* Protective Immunity against Severe Malaria in Children Is Associated with a Limited Repertoire of Antibodies to Conserved PfEMP1 Variants. *Cell Host Microbe* **26**, 579-590.e5 (2019).

31. Stahl, H.-D. *et al.* *Interspersed blocks of repetitive and charged amino acids in a dominant immunogen of Plasmodium falciparum (malaria/recombinant DNA/cDNA expression/colony immunoassay)*. *Immunology* vol. 82 (1985).

32. Lu, Y. *et al.* Chimeric peptide constructs comprising linear B-cell epitopes: Application to the serodiagnosis of infectious diseases. *Sci. Rep.* **5**, (2015).

33. Hou, N. *et al.* Low-Complexity Repetitive Epitopes of Plasmodium falciparum Are Decoys for Humoural Immune Responses. *Front. Immunol.* **11**, 610 (2020).

34. Longley, R. J. *et al.* Comparative assessment of vaccine vectors encoding ten malaria antigens identifies two protective liver-stage candidates. *Sci. Rep.* **5**, 1–13 (2015).
